# Identifying the Cause of Rupture of Li‐Ion Batteries during Thermal Runaway

**DOI:** 10.1002/advs.201700369

**Published:** 2017-10-27

**Authors:** Donal P. Finegan, Eric Darcy, Matthew Keyser, Bernhard Tjaden, Thomas M. M. Heenan, Rhodri Jervis, Josh J. Bailey, Nghia T. Vo, Oxana V. Magdysyuk, Michael Drakopoulos, Marco Di Michiel, Alexander Rack, Gareth Hinds, Dan J. L. Brett, Paul R. Shearing

**Affiliations:** ^1^ Electrochemical Innovation Lab Department of Chemical Engineering University College London Torrington Place London WC1E 7JE UK; ^2^ NASA Johnson Space Center Houston TX 77058 USA; ^3^ National Renewable Energy Laboratory 15013 Denver West Parkway Golden CO 80401 USA; ^4^ Diamond Light Source Harwell Science and Innovation Campus Didcot Oxfordshire OX110DE UK; ^5^ ESRF–The European Synchrotron 71 Rue des Martyrs 38000 Grenoble France; ^6^ National Physical Laboratory Hampton Road Teddington Middlesex TW11 0LW UK

**Keywords:** high‐speed imaging, Li‐ion batteries, thermal runaway, venting, X‐ray CT

## Abstract

As the energy density of lithium‐ion cells and batteries increases, controlling the outcomes of thermal runaway becomes more challenging. If the high rate of gas generation during thermal runaway is not adequately vented, commercial cell designs can rupture and explode, presenting serious safety concerns. Here, ultra‐high‐speed synchrotron X‐ray imaging is used at >20 000 frames per second to characterize the venting processes of six different 18650 cell designs undergoing thermal runaway. For the first time, the mechanisms that lead to the most catastrophic type of cell failure, rupture, and explosion are identified and elucidated in detail. The practical application of the technique is highlighted by evaluating a novel 18650 cell design with a second vent at the base, which is shown to avoid the critical stages that lead to rupture. The insights yielded in this study shed new light on battery failure and are expected to guide the development of safer commercial cell designs.

## 1 Introduction

The uptake of electric and hybrid electric vehicles (EVs and HEVs) brings forth a rapid increase in the demand for lithium‐ion batteries alongside an acute need for improvements in their safety and performance.^1^ As the energy density of batteries rises, their safety and reliability becomes increasingly important. When exposed to abusive electrical, thermal, or mechanical conditions, the active materials within lithium‐ion batteries break down exothermically, generating large amounts of heat which can lead to thermal runaway, accompanied by fire and/or explosion.^2^, ^3^, ^4^ Although the risk of a single cell failing is extremely low, the risk of cell failure within an EV becomes significant when thousands of cells are encased in a pack. In particular, the occurrence of a single cell undergoing thermal runaway causes great concern since cell‐to‐cell propagation of thermal runaway and the hazardous destruction of the entire system can occur.^5^

The 18650 cell (designated by it having a cylindrical geometry with a diameter of 18 mm and a length of 65 mm) is the most ubiquitous lithium‐ion cell geometry and one that is used in some EVs as well as other demanding applications such as human spaceflight.^6^ The capacity of 18650 cells has steadily been increasing since their initial production,^7^ with commercial cells today achieving >3 Ah. With increasing capacity, the rate and quantity of heat and gas generated during failure of the cells are also expected to rise. In a report from Sandia National Labs, Orendorff et al.^8^ measured a heating rate of 70 W Ah^−1^, a heat output of 9.1 kJ Ah^−1^, and a gas output of 2.5 L Ah^−1^ during thermal runaway of cylindrical 3.4 Ah LiNi_0.8_Co_0.15_Al_0.05_O_2_ (NCA) cells. The increasing heating rate, as well as heat and gas output, present engineering challenges to mitigate the risk of catastrophic failure. For example, gas generation within the rigid casing of an 18650 cell causes it to effectively become a pressure vessel, and if the pressure within the cell is not relieved in a controlled manner, then the cell can undergo violent rupture in the form of an explosion.

Integrated safety devices^9^ such as pressure relief vents, positive temperature coefficient (PTC) devices, current limiting switches, and current interrupt devices (CIDs) are designed to prevent the build‐up of gas, and if necessary, safely relieve pressure. However, a rapid rise in pressure can sometimes circumvent the safety mechanisms of even the most advanced cell designs. For example, as shown in a previous study,^4^ an 18650 cell can rupture if the pressure relief is hindered by the vent becoming clogged; this leads to the spread of hot projectiles and molten materials into the surrounding area. The hazards associated with such projectiles are widely recognized, and some testing standards, such as UL 2054,^10^ involve projectile tests for cells undergoing thermal runaway. The efficacy of the venting mechanism determines the risk of the cell exploding and producing projectiles, as well as playing an important role in heat dissipation of the cell during failure.^11^ A high rate and large volume of gas generation can lead to flares,^12^ which challenge the mechanical integrity of cells and their integrated safety devices. Therefore, it is imperative that the engineering design of cells evolves alongside their increasing energy density, and that the increasing risks associated with failure are understood and mitigated. This necessitates an applied understanding of battery failure with detailed empirical descriptions of the events that occur leading up to and during catastrophic failure,^13^ linking phenomena that occur within cells to risks posed externally. However, due to the complexity and high speed of catastrophic failure, as well as the myriad of possibilities for failure propagation, there remains limited understanding of the internal events that lead to cell rupture, limiting design options for preventing such phenomena.

X‐ray radiography and computed tomography (CT) have been used to noninvasively inspect internal architectures of materials and devices. Time‐lapse X‐ray imaging can be used to track the evolution of materials as they change over time, which has emerged as a highly effective method for characterizing degradation mechanisms of lithium‐ion batteries across multiple time and length scales.^3^, ^4^, ^14^ As shown in a previous study,^4^ thermal runaway can propagate throughout 18650 cells in <2 s, requiring a temporal resolution of >1000 frames per second (fps) to capture sufficient detail to describe the dynamic failure mechanisms. The process of cell rupture was observed to occur over <0.01 s, requiring a much higher temporal resolution and frame rate to achieve enough detail to characterize the process. X‐ray sources with an extremely high photon flux such as synchrotrons are required^15^ to achieve such high‐speed imaging capabilities. For example, recently a team at the Swiss Light Source (SLS) achieved high‐speed X‐ray CT at 20 Hz,^16^ and at the European Synchrotron (ESRF) the dynamics of an electric arc ignition in a fuse were captured via ultra‐high‐speed radiography at MHz frame rates.^17^

In this work, the high‐speed imaging capability of the ESRF^18^ has been used to image the venting and rupture mechanisms of five different commercial cell designs undergoing thermal runaway induced by thermal abuse. The rapid processes that lead to cell bursting are identified via high‐speed imaging at unprecedented rates of up to 20 272 fps. This approach allows the stages that lead to the most catastrophic type of failure (cell bursting) to be characterized, as well as more moderate types of failure. The insights achieved from the experiments performed at the ESRF clarify the need for cell designs with more effective pressure relief. The behavior of a prototype cell with a double‐vent design is then assessed via follow‐on high‐speed imaging experiments at the Diamond Light Source (DLS) synchrotron.^19^ The venting mechanisms of six different 18650 cell designs are compared with respect to safety, and the merits of each design, as well as suggestions for further improvements, are discussed.

## 2 Results and Discussion

### 2.1 18650 Cell Designs

The venting mechanisms of five different commercial 18650 cell designs and one prototype cell design were examined via high‐speed radiography during thermal runaway. The specifications of the tested cells are presented in **Table** **1**, where the cells contain one or more of four different electrode materials, LiNi*_x_*Mn*_y_*Co*_z_*O_2_ (NMC), LiNi*_x_*Co*_y_*Al*_z_*O_2_ (NCA), LiMn_2_O_4_ (LMO), and LiCoO_2_ (LCO).

**Table 1:** Specifications of 18650 lithium‐ion cells used during high‐speed radiography experiments. The voltage at which the cells were tested, as well as whether the cell contained a mandrel, is also provided

Model	Positive electrode	Capacity [Ah]	Voltage [V]	Mandrel
LG ICR18650‐S3	NMC	2.2	4.2	No
LG ICR18650‐B4	NMC	2.6	4.2	Yes
Panasonic NCR18650B	NCA	3.4	4.2	Yes
Samsung INR18650‐25R	NCA	2.5	4.2	No
Sanyo UR18650ZY	LMO and LCO	2.6	4.2	Yes
Bottom‐vent cell	NCA	2.8	4.2	No

**Figure 1:**
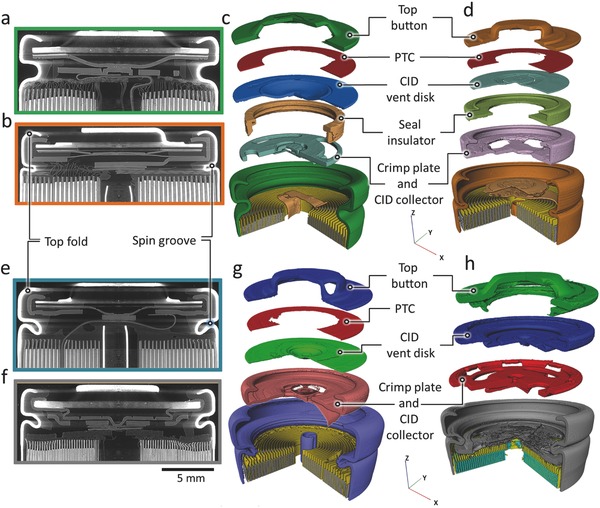
Greyscale *XZ* orthoslices from X‐ray CT reconstructions of a) LG‐S3 and b) Panasonic cells. Exploded 3D reconstructions of c) LG‐S3 and d) Panasonic cells, showing the placement of the integrated safety devices. Greyscale *XZ* orthoslices from e) Sanyo and f) Samsung cells. Exploded 3D reconstructions of g) Sanyo and h) Samsung cells, showing the placement of the integrated safety devices.

**Figure 2:**
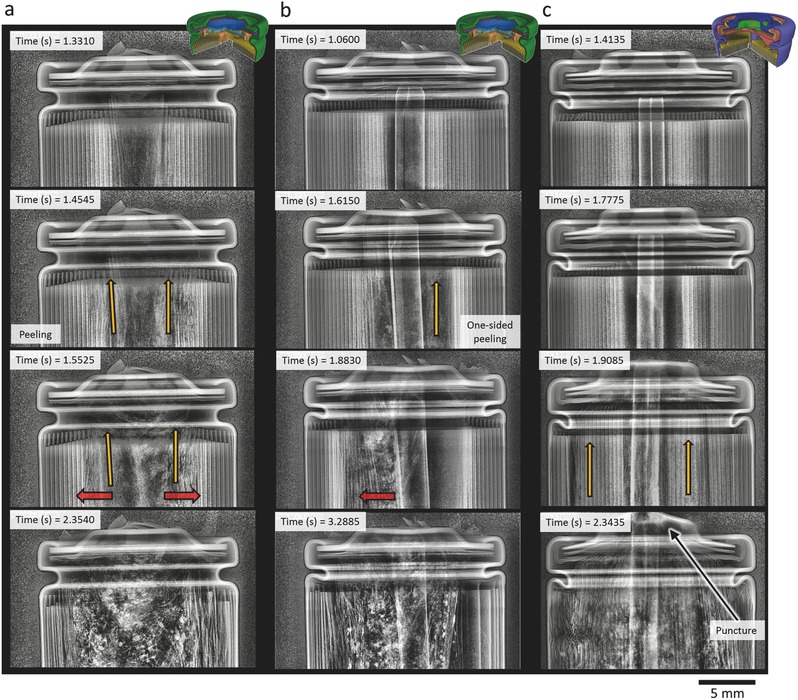
Time‐stamped radiographs of a) LG‐S3, b) LG‐B4, and c) Sanyo cells undergoing thermal runaway. Radiography movies of (a), (b), and, (c) are provided as Movies S1, S3, and S5, respectively, in the Supporting Information. The red arrows highlight the radial direction of propagation of thermal runaway, and the yellow arrows highlight the direction of peeling and shift of the electrode assembly.

The six cells represent a wide range of chemistries, capacities, and cell designs. The two LG cells, LG ICR18650‐S3 and LG ICR18650‐B4, will henceforth be referred to as LG‐S3 and LG‐B4. The vent region of each cell design was imaged using X‐ray CT (imaging conditions are provided in the following sections), and exploded 3D segmentations are presented in **Figure** **1**. The LG cells contained the same vent design (Figure 2a,c), but the LG‐B4 cell contained a cylindrical center mandrel which offered some safety advantages.^20^ Figure 2 shows the safety features and vent designs of the LG, Panasonic, Samsung, and Sanyo cells. The CID vent disk on each cell consisted of a conducting plate with a domed structure that was concave with respect to the electrode assembly. When the pressure inside the cell increased due to gas generation, the domed disk became convex and disconnected the circuit, preventing further discharge. In addition, each CID disk contained a scored annulus that ruptured when a certain pressure was reached, initiating the venting process. The LG, Panasonic, and Sanyo cells all contained PTC devices (Figure 2), in contrast to the Samsung cell due to it being designed for high‐rate discharge (20 A) applications. The PTC switch consisted of a conductive polymer layer between two metallic annular disks that greatly increased in electrical resistance at elevated temperatures.

The design of the spin groove of the crimp seal and its top fold of each cell, shown in the greyscale tomographic slices in Figure 2a,b,e,f, played an important role in the bursting process of each cell. The spin groove stretched as a result of rapid pressure rise during thermal runaway; its architecture determined the volume of gas that could accumulate within the cell before it burst and released the cell header, as well as the bursting pressure itself. The Panasonic cell contained the shortest spin groove height (Figure 1b). The Sanyo and Samsung cells had similar crimp designs with a deeper spin groove into the core of the cell, whereas the LG cells contained a shallower spin groove with a thick wall.

### 2.2 Controlled Ejection

The process of pressure relief determines the risks associated with cell failure. The crimp components are designed to relieve the pressure in a controlled way, with the release of hot and molten fluidized material in a known direction, and without high‐velocity projectiles. Internal cylindrical mandrels are designed to aid the release of pressure by providing a clear path for the fluidized material and gases to flow from the base to the crimp of the cell. As shown in a previous study,^4^ without an internal mandrel the electrode assembly can collapse into the vacant core which hinders the escape of gases and increases the risk of the cell bursting.

Three cell designs exhibited a well‐controlled pressure relief and fluid ejection process during thermal runaway with each cell design displaying characteristic failure mechanisms: the LG‐S3, the LG‐B4, and the Sanyo cells. The venting mechanism of each cell was captured by high‐speed radiography at 2000 fps with two repeat tests for the LG‐S3, LG‐B4, and Sanyo cells. In **Figure** **2**, selected radiographs from Movies S1, S3, and S5 in the Supporting Information are presented, and similar phenomena can also be seen in the movies in the Supporting Information of the repeat tests of the same cells (Movies S2, S4, and S6, Supporting Information).

**Figure 3:**
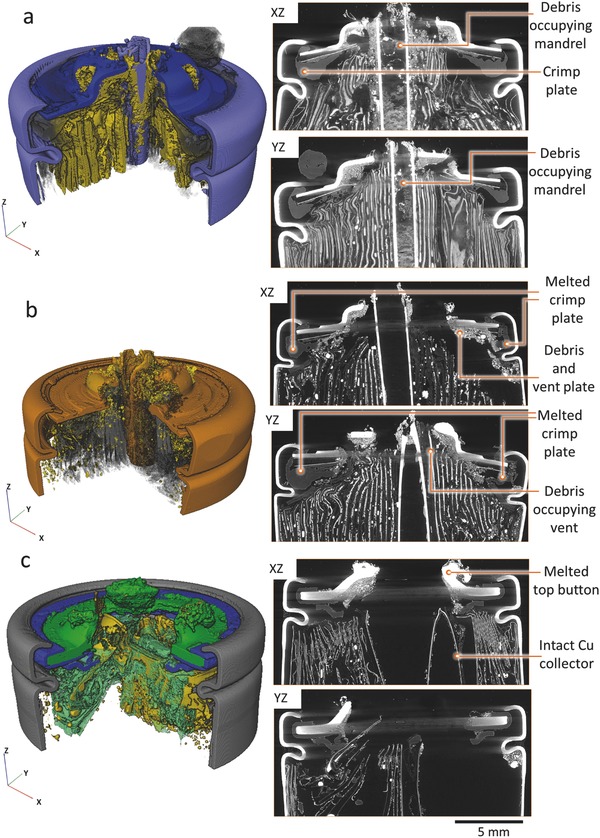
Postmortem 3D reconstructions and corresponding *XZ* and *YZ* orthoslices of a) Sanyo, b) Panasonic, and c) Samsung cells showing the damage to the top button after thermal runaway. The cylindrical mandrel in (a) and (b) is seen to protrude after puncturing the top button. The radiography movies showing the process of thermal runaway for the Sanyo, Panasonic, and Samsung cells are provided as Movies S5, S7, and S8 in the Supporting Information.

The LG‐S3 and LG‐B4 cells contained the same design of crimp components; however, the two cells differed in that the LG‐B4 cell contained an internal cylindrical mandrel and had a slightly higher capacity of 2.6 Ah. As highlighted by the red arrows in Figure 3a,b, the ejection process progressed radially outwards from the inner layers of the electrode assemblies of the LG‐S3 and LG‐B4 cells. The inner layers of the LG cell without the mandrel (LG‐S3) first shifted toward the vent, creating a V‐shape (as highlighted by yellow arrows at 1.4545 s in Figure 3a) and peeled away due to the shear force exerted by the escaping gas passing through the vacant core. Similarly, the inner layers of the electrode assembly of the LG‐B4 cells (with the mandrel) peeled away as shown after 1.6150 s in Figure 3b, but the gas flow caused the mandrel to shift to one side, which mitigated the peeling effect on the side on which the mandrel leaned, but accentuated the peeling on the opposite side.

In each of the three LG‐S3 cells, thermal runaway propagated radially outwards from the inner layers (shown at 1.5525 s in Figure 3a). The active materials of the electrode assembly broke down exothermically and began to fluidize. Large portions of the electrode assembly began to detach and eject, and in the case of the LG cell without the mandrel, some material accumulated beneath the top button of the cell, as shown between 1.5525 s and 2.3540 s in Figure 2a. Thermal runaway ran to completion within each of the three cells, with evidence of copper globules (highly attenuating white sections shown at 2.3540 s in Figure 3a), indicating that internal temperatures reached >1085 °C (the melting‐point of copper). However, the rate of propagation of thermal runaway in the radial direction varied between the three cells. As seen at 1.8830 s in Figure 3b, the ejection of a large portion of the electrode assembly on the right side of the mandrel interrupted the propagation of thermal runaway in the radial direction on that side.

### 2.3 Piercing by the Mandrel

The mandrel in both the LG‐B4 and Sanyo cells (Figure 3b,c) was propelled through the CID vent disk and crimp plate, and exerted significant force on the top button. The mandrel in both cells had a slit from bottom to top, which allowed gases to access the vacant core at any depth through the cell. The indent at the top of both mandrels allowed gas to escape the mandrel when pressed against the top button, providing a direct route for gases to escape from the base of the cell to the top. Furthermore, when compared to the failure mechanism of the LG‐S3 cell, the mandrel appears to have mitigated the build‐up of debris beneath the top button. However, in the Sanyo cell, the mandrel deformed the crimp components and punctured through the top button, as seen between 1.9085 s and 2.3435 s in Figure 3c.

The three cells described in Figure 3 remained intact during thermal runaway. There was no evidence that the cells approached their bursting pressure during thermal runaway as neither the top fold nor spin groove of the cells displayed any significant extension. However, a postmortem X‐ray CT image capturing the additional damage (piercing) to the top button of the Sanyo cell from the internal mandrel is shown in **Figure** **3**a.

**Figure 4:**
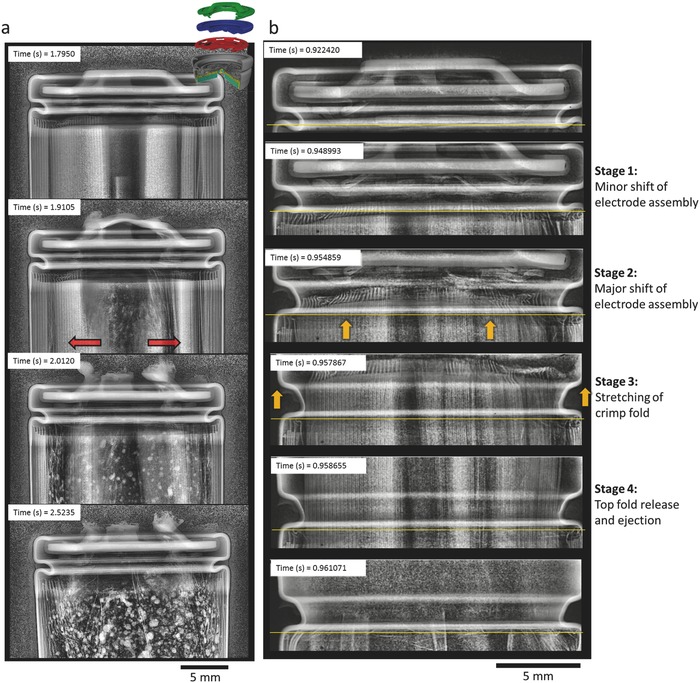
Time‐stamped radiographs taken at a) 2000 fps showing the propagation of thermal runaway within the Samsung cell and the top button melting, and b) taken at 20 272 fps showing the stages of the Samsung cell bursting. The thin yellow line on each image is a reference line at the shoulder of the spin groove. Radiography movies of (a) and (b) are provided as Movies S8 and S10, respectively, in the Supporting Information. The red arrows highlight the radial direction of propagation of thermal runaway, and the yellow arrows highlight the shift of the electrode assembly and extension of the spin groove.

The presence of debris and resolidified metals inside the cylindrical mandrel in Figure 4a indicates that the longitudinal slit in the mandrel allowed fluidized materials to pass through its hollow core from any depth within the cell, which would have helped mitigate peeling by reducing the shear force experienced by the inner layers of the electrode assembly during thermal runaway. The presence of copper globules within the mandrel indicates that the local temperature within the flow of material was >1085 °C, which when directed at the underside of the top button would weaken the steel and facilitate the mandrel puncturing through.

Similarly, the mandrel caused a puncture in the top button of the Panasonic cell (Figure 4b). Considering that the Panasonic mandrel weighed only 0.3 g, this indicates the more violent nature of thermal runaway in a cell that achieves >250 Wh kg^−1^ and >700 Wh L^−1^. The radiographs in Movie S7 in the Supporting Information show the lead‐up to the end‐state of the cell captured in the postmortem image of the Panasonic cell in Figure 4b. No debris was observed within the mandrel of the Panasonic cells, which did not contain a longitudinal slit. Consequently, the mandrel design within the Panasonic cell only relieved pressure from the base of the cell. By puncturing through the top button of the two cells, the mandrels provided a direct path for gases and fluidized materials to flow out of the casing. This is expected to have improved the pressure relief and reduced the risk of the cell bursting. However, a damaged top button would also increase the risk of the mandrel escaping as a high‐velocity projectile, which is seen in the following section.

The top button of the Samsung cell shown in Figure 4c (radiographs in Movie S8, Supporting Information) is suspected to consist of an alloy with a lower melting‐point than steel because the top button was observed to melt during thermal runaway, despite there being little presence of melted copper within the cell (indicating that most of the cell did not reach 1085 °C).

### 2.4 Cell Bursting and Mandrel Escape

Throughout all tests, the higher energy density cells were observed to be the most likely to burst. The increased likelihood is partially attributed to the higher capacity (3.4 Ah) relative to the rest of the cells (≤2.6 Ah) which, according the report by Orendorff et al.^8^ would produce an additional 2 L of gas during thermal runaway (based on ≈2.5 L Ah^−1^). The additional gas generation is also expected to result in increased force being applied on the internal mandrel and crimp components, irrespective of whether the vent clogged. The stages that lead up to bursting of the Samsung (2.5 Ah), LG (2.6 Ah), and Panasonic (3.4 Ah) cells were examined. Detailed imaging of the events surrounding the bursting process of an 18650 cell, which occurs over ≈0.01 s, is beyond the capability of imaging at 2000 fps (as demonstrated in Movie S9, Supporting Information where a Panasonic cell bursts). An increased frame rate of 20 272 fps was used to capture and characterize the bursting process, which required a reduction in the size of the field of view (FOV).

**Figure 5:**
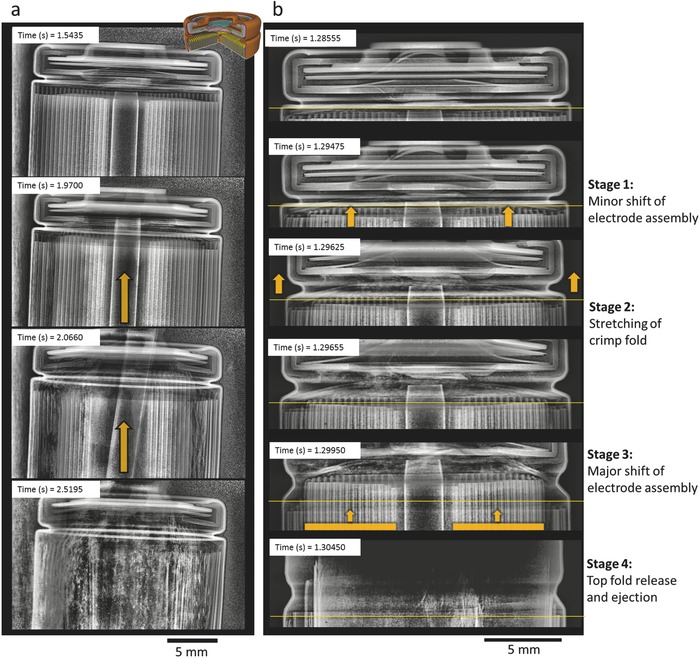
Time‐stamped radiographs taken at a) 2000 fps showing the mandrel piercing the crimp‐components during thermal runaway where the yellow arrows highlight the direction of shift of the mandrel, and b) taken at 20 272 fps showing the stages of the 18650 Panasonic cell bursting. Radiography movies of (a) and (b) are provided as Movies S12 and S13, respectively, in the Supporting Information.

When exposed to the same setup and heating rate, two out of seven Samsung cells that were tested burst. The five cells that did not burst underwent a controlled release of pressure and ejection of broken‐down material. The ejection process was improved by the top button melting (Figure 4c) and clearing a path for the active material to eject. As highlighted by red arrows in **Figure** **4**a, thermal runaway propagated from the inner layers of the cell radially outwards. The flow of gas through the core of the cell carried the active material out through the top button as it broke down. In the case of the Samsung cells that burst, it appeared that the electrode assembly collapsed into its hollow core, hindered the escape of gas, and led to the ejection of the electrode assembly via a four‐stage process (Figure 5b):

- Stage 1 involved a minor shift of the electrode assembly toward the vent.
- Stage 2 involved a sudden major shift (highlighted by the yellow arrows in Figure 5b) of the electrode assembly toward the vent, clogging the vent and exerting force on the crimp components.
- Stage 3 involved the spin groove stretching out as a result of the force applied by the electrode assembly on the crimp components.
- Stage 4 involved the top fold straightening out and releasing the crimp components followed by ejection of the electrode assembly, completing the bursting process.

Here, the shift of the electrode assembly against the crimp components caused the vent to clog, which is likely to be the primary cause of the cell bursting. The collapse of the electrode assembly is thought to have led to the major shift, via a rapid build‐up of pressure at the base of the cell with no alternative path through which it could be relieved.

The LG‐B4 cell had a similar capacity (2.6 Ah) to the Samsung cell (2.5 Ah) and is expected to have generated a similar quantity of gas. However, the LG cell contained a cylindrical mandrel that supported the electrode assembly and prevented collapse of the assembly structure into its core, as seen previously.^4^ In Movie S11 in the Supporting Information, the electrode assembly and mandrel are also seen to have shifted toward the vent, exerting force on the crimp components and leading to ejection of the electrode assembly. The mandrel in the LG‐B4 cell moved independently from the electrode assembly, exerted force on and deformed the crimp components before the electrode assembly made contact. The movement of the mandrel through the core of the electrode assembly indicates that, despite the open pathway for the gas to flow from the base of the cell to the vent, the pressure relief from the base of the cell was still insufficient to prevent the assembly shifting toward and clogging the vent.

**Figure 6:**
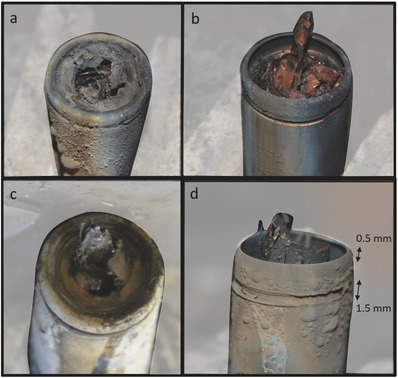
Postmortem photographs of the 18650 Samsung cells where a) the top button melted, and b) the cell burst, as well as the Panasonic cells that underwent c) piercing of the top button by the mandrel, and d) bursting.

The bursting mechanism of the Samsung and LG‐B4 cells appeared to stem from a pressure build‐up beneath the FOV that led to the electrode assembly shifting toward and clogging the vent. Within about 0.01 s of the electrode assembly making contact with the crimp components, the cell reached its burst‐pressure and released the crimp components. The Panasonic cell also contained a mandrel, but its capacity was more than 30% greater (3.4 Ah) than the Samsung and LG cells. Of the 11 Panasonic cells tested, 6 burst via spin groove release of the cell header, 4 underwent crimp‐puncture by the internal mandrel, and 1 stayed intact. **Figure** **5**a is extracted from Movie S12 in the Supporting Information and shows an example of the crimp‐components of the Panasonic cell being punctured by the internal mandrel, where the mandrel escaped as a projectile. Thermal runaway initiated 5 winds in from the outer casing (seen at 1.9700 s in Figure 6a), and the rapid peeling of the inner layers of the electrode assembly indicates a high flow rate of gas through the core. As highlighted by the yellow arrow at 2.0660 s in Figure 6a, the mandrel shifted position and was propelled up against the crimp components. Thereafter, the mandrel exerted enough force to pierce the top button and escape the cell at a high velocity. According to Orendorff et al.,^8^ a gas output of ≈8 L was measured during thermal runaway of similar cylindrical 3.4 Ah NCA cells. Generation of such a large volume of gas over a short time period would lead to a high flow rate of gas through the vent, and is thought to have caused the mandrel to propel against and exert force on the top button. Following the top button being pierced and the core of the electrode assembly being cleared, none of the cells proceeded to burst, which was due to the improved pressure relief.

The bursting process of the Panasonic cells also occurred in four stages (Figure 6b):

- Stage 1 involved a minor shift of the electrode assembly toward the vent. The portion of the assembly directly beneath the spin groove stayed in position, while the inner portion sheared away.
- Stage 2 involved the spin groove straightening out. This stage did not appear to be influenced by the force exerted by the shift of the assembly, but by the build‐up of gas beneath the crimp components. This provides evidence that the gas cannot escape at a sufficient rate, irrespective of the vent being clogged.
- Stage 3 involved a major shift of the electrode assembly and cylindrical mandrel toward the vent, thereafter exerting force on the crimp components.
- Stage 4 involved the final step of the top fold straightening out as a result of the force exerted by the electrode assembly, releasing the cell header. This was followedby the complete ejection of the electrode assembly, completing the bursting process.

The order of Stages 2 and 3 in the bursting process of the Panasonic cell indicates that it is not necessarily vent‐clogging alone that can lead to an 18650 cell bursting during thermal runaway, but a vent design that does not allow a sufficient flow rate of gas to escape. The spin groove of the Panasonic cell extended without force being applied by the electrode assembly or the mandrel. A pocket of gas formed between the crimp components and the electrode assembly while the spin groove continued to extend. This indicates that the vent design of the cell did not allow escape of a sufficient flow rate of gas to avoid reaching the pressure required to extend the spin groove. However, the top fold retained the crimp components until the electrode assembly underwent a major shift toward the vent (Stage 3 in Figure 6b), at which point the top fold extended and released the crimp components, and the electrode assembly ejected. This result highlights the need for a more effective pressure relief strategy for high capacity cells, where large quantities of gas can be generated over a very short time during thermal runaway.

### 2.5 Considerations for Module Design

Postmortem photographs of the Samsung and Panasonic cells described in Figure 5 and Figure 6 are provided in **Figure** **6**. The occurrence of bursting, mandrel piercing, and melting of the top button poses different risks when the 18650 cells are fitted as part of a battery pack or module assembly. For example, extension of the spin groove and straightening of the top fold necessitate space above the vent end of the 18650 cell. In the case of the Samsung and Panasonic cells in Figure 6b,d, the extended spin groove and top fold added ≈1.5 and 0.5 mm respectively, to the total length of the cells. Therefore, a 2 mm allowance for the cell to extend in the longitudinal direction is required for the bursting process to occur. Preventing the extension of the folds is likely to increase the risk of side‐wall rupture, a phenomenon that is widely known to significantly increase the risk of cell‐to‐cell propagation of thermal runaway. An example of how the extension of the spin groove and top fold might be hindered is by an end‐plate or mounting‐bracket in a battery module that applies compressive force to the top and base of the cells.

The projectiles from the pierced and burst cells contained enough momentum to reach several meters from the cell. Such hot projectiles can pose great risks, particularly for transport and shipping, where flammable materials might be present. Similarly, the flare of hot and molten material from the section of the melted cap could lead to rapid heat transfer and cause propagation of thermal runaway to neighboring cells, if not configured safely in modules. Conversely, if directed toward a safe location, hot flares may help dissipate the heat from failing cells and reduce the risk of cell‐to‐cell propagation.

### 2.6 Application of a Second Vent

**Figure 7:**
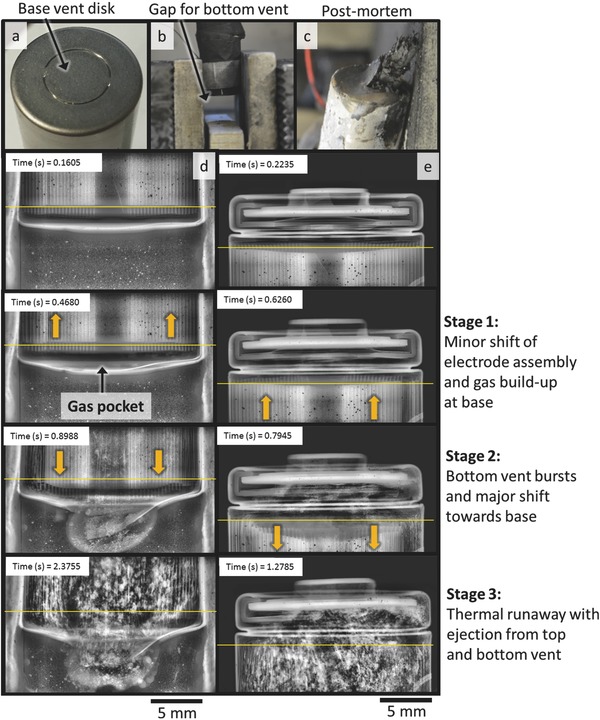
a) Photograph showing the vent disk at the base of the 18650 prototype cell. b) Photograph showing the position of the 18650 cell with clearance at its base for the second vent. c) Postmortem photograph showing ruptured vent disk. d) Time‐stamped radiographs showing the activation of the secondary vent during thermal runaway. e) Time‐stamped radiographs showing the crimp‐region of the 18650 cell during thermal runaway. Radiography movies of (d) and (e) are provided as Movies S14 and S16, respectively, in the Supporting Information.

As previously observed, high energy density cells can produce gas at a sufficiently high rate to cause a pressure rise that extends the spin groove and leads to bursting without the vent being clogged by the electrode assembly. This demonstrates a clear need for more effective venting mechanisms to coincide with the increasing energy density of cells. One such strategy is the incorporation of a second vent at the base of the cell and, in light of this observation, a high energy density (2.8 Ah) 18650 prototype cell with a secondary vent at its base (**Figure** **7**a) was examined. The base vent consisted of a scored disk in the casing with two nonweakened sections that hold the disk in place when it ruptures (Figure 1a). The cell was held in mid‐air in the upright position by hydraulic clamps, with a gap beneath the cell that allowed the base vent to activate (Figure 1b). During activation, the disk ruptured and folded back on the hinge allowing release of the contents of the cell (Figure 1c).

Five cells with base vents were tested, all of which showed similar behavior. Movies S14 and S15 in the Supporting Information show the bottom vent activating, and the events that occur at the top vent for one cell are shown in Movie S16 in the Supporting Information. In each of the cells the ejection process occurred in three stages:

- Stage 1 involved a minor shift of the electrode assembly toward the top vent (highlighted by the upward pointing arrows in Figure 1d,e). This corresponded with a pocket of gas forming at the base of the cell and the base plate expanding out into a dome shape.
- Stage 2 involved the bottom vent activating and the electrode assembly undergoing a major shift toward the base of the cell (highlighted by the downward pointing arrows in Figure 1d,e).
- Stage 3 involved the controlled release of the broken‐down active materials from the top and bottom vents of the cell. There was no evidence of the spin groove or top fold extending or side wall rupture, indicating that the double‐vent design provided effective pressure relief during thermal runaway.

The initial stage of the thermal runaway process resembled that of the single‐vent cells, where a minor shift of the electrode assembly toward the top vent occurred. The doming of the base of the cell at this point indicates that pressure built up beneath the electrode assembly, which caused a minor shift of the assembly upwards toward the top vent. The primary path through which the pressure at the base of the cell could be relieved was through the core of the assembly toward the top vent. However, unlike the single‐vent cells, the pressure was relieved from the base of the cell through the base vent, rather than relying on a clear path through the electrode assembly. The pressure at which the bottom vent activated was much lower than that which was required for the spin groove to extend and the cell to burst. This helped prevent a major shift of the electrode assembly toward the vent, extension of the spin groove, and ultimately the cell bursting.

The electrode assembly shifted toward the base vent during the second stage of the thermal runaway process (Figure 1d,e). This indicates that greater pressure relief was provided by the bottom vent than the top vent. Thermal runaway propagated relatively slowly throughout the cell and the broken‐down material was ejected from both ends. In contrast to the single‐vent cells examined previously, peeling of the inner layers of the electrode assembly did not appear to occur in the double‐vent cells. This was because there was no pressure rise at the base of the cell, reducing the directed stream of gas toward the top vent and therefore the shear on the inner layers.

By providing an alternative escape route for generated gas, the base‐vent design appeared to reduce the risk of cell rupture and projectile generation. Furthermore, the role of the internal cylindrical mandrel in maintaining an open path for gases to flow from the base to the top vent of the cells is made redundant with the introduction of the base vent; the risk of a collapsed electrode assembly leading to the vent being clogged no longer exists. However, the base‐vent design and the inherent ejection of hot material from both ends of the 18650 cell during thermal runaway may require a new approach to the design of battery modules, such as additional space being provided below the cells, which would come at the expense of the volumetric energy density of the module.

## 3 Conclusions

The combined use of high‐speed X‐ray radiography and CT has been shown to be a powerful tool for linking external risks posed by 18650 lithium‐ion cells during thermal runaway with internal phenomena. The venting mechanisms of five different 18650 cell designs from four leading manufacturers were analyzed, and the occurrences that lead to four types of failure were characterized: controlled ejection of contents, cell bursting, puncture of the top button, and escape of the internal mandrel. The internal mandrel was shown to support the electrode assembly and provide a clear path through which fluidized broken‐down materials can escape via the vent during thermal runaway. The apparent drawback of the mandrel is that high flow rates of gas can lead to the mandrel shifting independently from the electrode assembly and sometimes puncturing the crimp components. In high‐capacity cells, where greater quantities of gases are generated, the mandrel can achieve enough momentum to pierce through the top button and become a high‐velocity projectile outside the cell which can be a major hazard in battery systems and during shipping. To avoid the mandrel being released as a projectile, it could be fixed to the base plate of the cell or contain a flattened end next to the vent that reduces the risk of puncture.

Using high‐speed X‐ray imaging, the lead‐up to cell rupture was captured in unprecedented detail and the bursting process was characterized as occurring in distinct stages. Two causes of bursting were identified. First, bursting occurs when the electrode assembly does not maintain an opening to allow sufficient gas flow to reach the vent, causing the electrode assembly to shift and clog the vent, which was the leading cause of cell rupture in this study. Second, when the vent itself does not allow sufficient flow of gas to escape, the internal pressure can rise to reach the cell's burst‐pressure. The second mechanism is more likely to occur in high‐capacity cells where a much greater quantity of gas is generated during thermal runaway. The two causes of bursting described here were prevented by the inclusion of a second vent at the base of the 18650 cell, which quickly relieved pressure before the spin groove could extend or the electrode assembly could shift and clog the vent. Pressure relief at both ends of the 18650 cell rendered the mandrel redundant for the purpose of maintaining an open channel for gases to escape. Therefore, the inclusion of a base vent improves the ability to safely manage rapid gas generation during thermal runaway and greatly reduces the risk of cell rupture.

In summary, high‐speed X‐ray imaging has facilitated evaluation of the effectiveness of safety devices and mechanical designs of commercial cells in mitigating risks associated with thermal runaway. It is important to note that the cells in this work were intentionally exposed to extreme thermal conditions that are certain to lead to thermal runaway. These conditions are exceedingly rare during normal operation “in‐field” scenarios. The insights discussed here have highlighted the need for mechanical designs of commercial cells to evolve alongside their increasing energy and power densities, especially since failure of high‐performance cells is often accompanied by high‐risk consequences through increased heat and gas production. This study demonstrates the potential for linking other risks, such as side‐wall rupture, total heat output, and the rate of gas generation, with internal dynamics of cells during failure. It is expected that this diagnostic approach is a precedent for further analyses that combine multiple metrics with the aim of developing a comprehensive description of battery failure and associated risks.

## 4 Experimental Section

**Figure 8:**
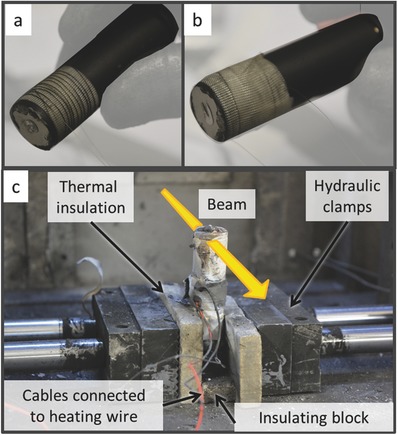
a) NiCr wire with a length corresponding to a resistance of ≈22 Ω wrapped around the base of an 18650 cell. The wire is separated from the cell casing by glass cloth tape. b) An additional layer of glass cloth tape was wrapped around the NiCr wire to prevent short circuiting. c) A failed 18650 cell secured in place by hydraulic clamps and insulation plates.

*Experimental Setup*: The experiments were performed at beamlines ID19 and I12 of the ESRF and DLS synchrotrons, respectively. The 18650 cells were heated by passing a current through high‐resistance NiCr wire wrapped around the base of the cells (**Figure** **8**a,b). The NiCr wire was cut to a length that gave a resistance of around 22 Ω and a suitable current to generate a heating power of 33 W was applied through the use of a power supply. High‐temperature glass cloth tape was wrapped above and below the heating coil to avoid short circuiting (Figure 8a,b).

The 18650 cells underwent thermal runaway in an explosion‐proof chamber with integrated X‐ray transparent windows (a description and photographs of the entire system are provided as Supporting Information with ref. (21)). The 18650 cells, with the heating coil applied were secured in line with the X‐ray beam by hydraulic clamps (operated at a pressure of 3 bar) with intermediate ceramic insulation plates to minimize heat loss to the metallic clamps (Figure 8c). A 16 mm wide piece of insulation plate was placed underneath the cell to avoid the clamps crushing the cell casing during thermal runaway.

*X‐Ray Computed Tomography*: 3D tomograms of the vent region of all the cells were taken at the ID19 beamline of the ESRF (Figure 2). The cells were imaged using a polychromatic beam with a 9.65 mm × 15.20 mm (horizontal × vertical) FOV, which consisted of 1302 × 2048 pixels with a pixel resolution of 7.42 × 10^−6^ m. The horizontal dimension of the FOV amounted to half of the 18650 cell; the cell was positioned such that its radial center was at the edge of the FOV, and therefore a 360° rotation enabled the entire cell to be captured and reconstructed.^22^ Each tomogram consisted of 3600 images with angular increments of 0.1°, each with an exposure time of 0.2 s. A PCO.Dimax (PCO AG, Germany) detector lens coupled with an LuAG:Ce (Ce‐doped Lu_3_Al_5_O_12_) scintillator was used, and the tomograms were reconstructed using a standard filtered back projection (FBP) algorithm.^23^

*High‐Speed Radiography*: To capture the dynamic occurrences during thermal runaway, a range of imaging conditions were utilized. **Table** **2** presents the imaging conditions for three different frame rates. At the ESRF, a polychromatic beam with a peak energy of 75 keV and a high‐speed PCO.Dimax camera (PCO AG, Germany) were used for all imaging experiments. Two scintillators were used during the imaging experiments: a GGG:Eu (Eu‐doped Gd_3_Ga_5_O_12_) and an LuAG:Ce scintillator, as listed in Table 2. The PCO.Dimax camera had 36 GB of on‐board memory for fast intermediate storage. A postevent triggering technique was used for recording, whereby the camera recorded continuously until triggered, at which point the previous 36 GB of data was stored and uploaded to the ESRF central servers. The time period corresponding to 36 GB of data collection depended on the frame rate and size of the FOV whereby for a predetermined frame rate, a larger FOV was more data‐intensive and consequently had shorter recorded time periods. Typically, the time period of interest was cropped to ≈3 s surrounding thermal runaway. A summary of frame rates and FOVs used in this work are presented in Table 2.

**Table 2:** Imaging conditions at the ESRF and DLS for different frame rates

Source	Frame rate [s^−1^]	Exposure [µs]	FOV [pixels]	Pixel [µm]	Energy [keV]	Scintillator
ESRF	2000	460	2016 × 1292	11.35	Poly	GGG:Eu
DLS	2000	490	1280 × 800	17.90	74	LuAG:Ce
ESRF	16 742	20	1008 × 248	22.70	Poly	LuAG:Ce
ESRF	20 272	40	816 × 236	22.70	Poly	LuAG:Ce

Experiments performed at DLS utilized a Vision Research Phantom Miro 310 high‐speed imaging camera (Vision Research, NJ, USA), and the high‐speed imaging experiments at 2000 fps were performed using a monochromatic 74 keV beam and an LuAG:Ce scintillator (Table 2).

To demonstrate reproducibility and provide confidence in the results, several repeat tests were carried out for each cell design. For brevity, 16 movies consisting of high‐speed X‐ray imaging of failing cells are provided as Supporting Information, which include one repeat test for each phenomenon described. For reference, a summary of the cell type, imaging rate, and failure mechanisms is provided in **Table** **3**. The failure mechanisms mentioned in Table 3 refer to the range of failure events observed, namely, when thermal runaway causes venting but is completely contained within the 18650 casing with no rupture (Contained), when the internal mandrel pierces through the top button but stays within the cell (Pierced), when the internal mandrel punctures the top button and escapes as a projectile (Escape), and when the cell bursts and releases its crimp components (Burst). The bottom‐vent cells underwent an independent failure scenario where a modified 18650 cell design with a second bursting disk at its base activated (2nd vent). Each of the respective failure events are discussed in detail in the Results and Discussion section.

**Table 3:** Summary table of the cell type, imaging rate, and failure mechanism contained within each of the Supporting Information Movies

Supporting Information Movie	Cell type	Imaging [fps]	Failure mechanism
1	LG‐S3	2000	Contained
2	LG‐S3	2000	Contained
3	LG‐B4	2000	Contained
4	LG‐B4	2000	Contained
5	Sanyo	2000	Pierced
6	Sanyo	2000	Contained
7	Panasonic	2000	Pierced
8	Samsung	2000	Contained
9	Panasonic	2000	Burst
10	Samsung	20 272	Burst
11	LG‐B4	20 272	Burst
12	Panasonic	2000	Escape
13	Panasonic	16 742	Burst
14	Bottom vent	2000	Second vent
15	Bottom vent	2000	Second vent
16	Bottom vent	2000	Second vent

## Conflict of Interest

The authors declare no conflict of interest.

## Supporting information

Supplementary

Supplementary

Supplementary

Supplementary

Supplementary

Supplementary

Supplementary

Supplementary

Supplementary

Supplementary

Supplementary

Supplementary

Supplementary

Supplementary

Supplementary

Supplementary
